# Supplementary material for: Empirical methods for controlling false positives and estimating confidence in ChIP-Seq peaks
Source: BMC Bioinformatics. 2008 Dec 5;9:523. doi: 10.1186/1471-2105-9-523 (PMC2628906; doi:10.1186/1471-2105-9-523)
Supplement: Additional File 1 — A variety of html documents from the USeq web site detailing the available applications, their best usage, output file type descriptions, command line menus, etc. [file 1471-2105-9-523-S1.zip › USeqUserGuides/JavaDoc.zip!/JavaDoc/util/bio/annotation/GeneGroup.html]

GeneGroup


|  |  |  |
| --- | --- | --- |
| **Overview** \| **Package** \| **Class** \| **Use** \| **Tree** \| **Deprecated** \| **Index** \| **Help** | |  |
| **PREV CLASS**   **NEXT CLASS** | **FRAMES**    **NO FRAMES**     **All Classes** |  |
| SUMMARY: NESTED \| FIELD \| CONSTR \| METHOD | DETAIL: FIELD \| CONSTR \| METHOD |  |


---


## util.bio.annotation Class GeneGroup

```
java.lang.Object
  util.bio.annotation.GeneGroup
```

**All Implemented Interfaces:**: java.io.Serializable, java.lang.Comparable

---

``` public class GeneGroup extends java.lang.Object implements java.lang.Comparable, java.io.Serializable ```

A GeneGroup is the top organizing level of a "Gene". It may contain multiple transGrps
(one transcript, possibly a translation, each with one or more exons) and a composite/ summary GeneRep (a gene representation broken into
coding and non-coding regions) .
Thus GeneGroup -> one GeneRep + one or more TransGroups -> transcript, exon, introns....

**See Also:**: Serialized Form

---

**Constructor Summary**

`GeneGroup(java.lang.String name,
java.lang.String chromosome,
int start,
int end,
java.lang.String type,
TransGroup[] transGrps,
int orientation,
java.lang.String attributes)`


| **Method Summary** | |
| --- | --- |
| `static java.lang.String` | `ALToString(java.util.ArrayList al)` |
| `int` | `compareTo(java.lang.Object other)` |
| `static java.lang.String` | `convertNumOrientationToStrand(int orientation)` |
| `static int` | `convertPlusToNumOrientation(java.lang.String orientation)` |
| `static java.util.ArrayList` | `extractStartEnds(ExonIntron[] exons)` |
| `static int[][]` | `extractStartEndsInts(ExonIntron[] exons)` |
| `static java.util.ArrayList` | `fetchIntrons(java.util.ArrayList exons)`             Takes an ArrayList of exon startStop int[2]'s, returns an ArrayList of int[2]'s representing the start,stop for each intron |
| `java.lang.String` | `fetchSumaryLine()` |
| `java.lang.String` | `getAttributes()` |
| `java.lang.String` | `getChromosome()` |
| `int` | `getEnd()` |
| `GeneRep` | `getGeneRep()` |
| `java.lang.String` | `getLabel()` |
| `java.lang.String` | `getName()` |
| `int` | `getOrientation()` |
| `int` | `getStart()` |
| `TransGroup[]` | `getTransGrps()` |
| `java.lang.String` | `getType()` |
| `static java.util.ArrayList` | `joinSegments(java.util.ArrayList segs)`             Takes an ArrayList of int[2]'s representing start stop coords kills the dups, and those cotained within another seg, will merge overlapping segments. |
| `void` | `setOrientation(int orientation)` |
| `static java.util.ArrayList` | `sortSegments(java.util.ArrayList segs)`             Sorts an ArrayList of int[2]'s representing start, end |
| `java.lang.String` | `toString()` |

**Methods inherited from class java.lang.Object**

`equals, getClass, hashCode, notify, notifyAll, wait, wait, wait`

**Constructor Detail**

### GeneGroup

```
public GeneGroup(java.lang.String name,
                 java.lang.String chromosome,
                 int start,
                 int end,
                 java.lang.String type,
                 TransGroup[] transGrps,
                 int orientation,
                 java.lang.String attributes)
```


**Method Detail**

### compareTo

```
public int compareTo(java.lang.Object other)
```

:   **Specified by:**: `compareTo` in interface `java.lang.Comparable`

---


### fetchSumaryLine

```
public java.lang.String fetchSumaryLine()
```

---


### toString

```
public java.lang.String toString()
```

:   **Overrides:**: `toString` in class `java.lang.Object`

---


### getOrientation

```
public int getOrientation()
```

---


### getGeneRep

```
public GeneRep getGeneRep()
```

---


### getTransGrps

```
public TransGroup[] getTransGrps()
```

---


### getLabel

```
public java.lang.String getLabel()
```

---


### getChromosome

```
public java.lang.String getChromosome()
```

---


### getAttributes

```
public java.lang.String getAttributes()
```

---


### getName

```
public java.lang.String getName()
```

---


### getEnd

```
public int getEnd()
```

---


### getStart

```
public int getStart()
```

---


### convertPlusToNumOrientation

```
public static int convertPlusToNumOrientation(java.lang.String orientation)
```

---


### convertNumOrientationToStrand

```
public static java.lang.String convertNumOrientationToStrand(int orientation)
```

---


### extractStartEndsInts

```
public static int[][] extractStartEndsInts(ExonIntron[] exons)
```

---


### extractStartEnds

```
public static java.util.ArrayList extractStartEnds(ExonIntron[] exons)
```

---


### fetchIntrons

```
public static java.util.ArrayList fetchIntrons(java.util.ArrayList exons)
```

:   Takes an ArrayList of exon startStop int[2]'s, returns an ArrayList of int[2]'s representing
    the start,stop for each intron

---


### ALToString

```
public static java.lang.String ALToString(java.util.ArrayList al)
```

---


### sortSegments

```
public static java.util.ArrayList sortSegments(java.util.ArrayList segs)
```

:   Sorts an ArrayList of int[2]'s representing start, end

---


### joinSegments

```
public static java.util.ArrayList joinSegments(java.util.ArrayList segs)
```

:   Takes an ArrayList of int[2]'s representing start stop coords
    kills the dups, and those cotained within another seg, will
    merge overlapping segments.

---


### getType

```
public java.lang.String getType()
```

---


### setOrientation

```
public void setOrientation(int orientation)
```


---


|  |  |  |
| --- | --- | --- |
| **Overview** \| **Package** \| **Class** \| **Use** \| **Tree** \| **Deprecated** \| **Index** \| **Help** | |  |
| **PREV CLASS**   **NEXT CLASS** | **FRAMES**    **NO FRAMES**     **All Classes** |  |
| SUMMARY: NESTED \| FIELD \| CONSTR \| METHOD | DETAIL: FIELD \| CONSTR \| METHOD |  |


---
